# Regional anesthesia combined with esketamine analgesia and remimazolam sedation as a salvage strategy for intertrochanteric femur fracture surgery in a high-risk patient: A case report

**DOI:** 10.1097/MD.0000000000049778

**Published:** 2026-07-10

**Authors:** Xiaolin Wang, Jingjing Zhang, Jing Ren, Ya Liu, Yanchao Yang

**Affiliations:** a Department of Anesthesiology, Shijiazhuang People’s Hospital, Shijiazhuang, Hebei, China.

**Keywords:** anesthesia contraindications, fascia iliaca compartment block, intertrochanteric fracture, multimodal anesthesia, ultrasound-guided nerve block

## Abstract

**Rationale:**

The anesthetic management of intertrochanteric fractures in patients with dual contraindications to neuraxial and general anesthesia is highly challenging. We illustrate a unique salvage multimodal strategy combining a comprehensive ultrasound-guided regional blockade with esketamine-based sedation to manage such complex cases.

**Patient concerns:**

A 55-year-old female (147 cm, 45 kg) was admitted following a slip-and-fall accident resulting in severe right hip pain and limited mobility. Due to her complex medical history, the patient and her family expressed significant anxiety regarding the risks of conventional anesthesia.

**Diagnoses:**

Imaging confirmed a comminuted right intertrochanteric fracture. The patient’s clinical profile was complicated by congenital spinal dysraphism (spina bifida) with a local meningocele and a history of 2 recent episodes of spontaneous pneumothorax.

**Interventions:**

The patient underwent surgical fixation under a primary ultrasound-guided supra-inguinal fascia iliaca compartment block (40 mL of 0.4% ropivacaine) supplemented by a lateral femoral cutaneous nerve block. Intraoperative management utilized a multimodal approach combining esketamine-mediated systemic analgesia and remimazolam-based sedation while strictly preserving spontaneous ventilation.

**Outcomes:**

The 70-minute procedure was successful without conversion to general anesthesia or positive-pressure ventilation. Hemodynamics remained stable throughout the surgery (systolic blood pressure: 128–151 mm Hg; heart rate: 61–78 bpm). The patient awakened fully within 3 to 4 minutes postoperatively, reported no intraoperative discomfort, and achieved excellent pain control (VAS 1/10) with an unremarkable one-month recovery.

**Lessons:**

For high-risk surgical candidates where both neuraxial and general anesthesia are relatively contraindicated, a multimodal approach centered on comprehensive nerve blocks combined with remimazolam and esketamine sedation represents a safe and effective salvage strategy. This technique minimizes physiological stress and avoids the specific risks of conventional anesthesia in complex clinical scenarios.

## 1. Introduction

Intertrochanteric fractures are a predominant injury in the elderly, often resulting from low-energy falls and requiring surgical fixation to enable early mobilization and reduce mortality.^[1]^ The choice of anesthetic technique is crucial, as these patients frequently present with significant comorbidities that increase perioperative risk. Neuraxial anesthesia and general anesthesia are the conventional options, yet each carries specific contraindications. Neuraxial techniques may be precluded by conditions such as congenital spinal dysraphism, while general anesthesia poses heightened risks for patients with a history of recurrent pneumothorax or severe respiratory compromise.^[2,3]^ This clinical challenge necessitates the exploration of safe and effective alternative anesthetic protocols for these high-risk surgical candidates.

The advent of ultrasound guidance has revolutionized regional anesthesia, facilitating the use of peripheral nerve blocks as a primary surgical anesthetic.^[4]^ The fascia iliaca compartment block (FICB), particularly the supra-inguinal approach, effectively anesthetizes the major innervation of the hip, including the femoral, lateral femoral cutaneous, and obturator nerves.^[5]^ However, as a sole anesthetic, it may not always suffice due to anatomical variability or inadequate sedation. Consequently, a multimodal strategy combining a comprehensive FICB with light intravenous sedation has been proposed to harness the benefits of regional anesthesia – such as superior analgesia and hemodynamic stability – while ensuring patient comfort and overcoming the limitations of a pure block technique.^[6]^

We herein present a case of a 55-year-old woman with relative contraindications to both neuraxial and general anesthesia who successfully underwent surgical repair of a right intertrochanteric fracture under a novel multimodal anesthetic. This approach utilized an ultrasound-guided supra-inguinal FICB as the cornerstone, supplemented with esketamine analgesia and remimazolam-based sedation as part of a multimodal regimen. This report aims to illustrate the feasibility and advantages of this technique, suggesting it as a viable primary anesthetic strategy for complex patients in whom standard approaches are undesirable.

## 2. Case report

### 2.1. Patient history

A 55-year-old woman (height: 147 cm, weight: 45 kg) was admitted on September 5, 2025, presenting with right hip pain and limited mobility for 10 days after a slip-and-fall accident where she landed on her right hip. The pain was immediate and persistent. A computed tomography (CT) scan confirmed a comminuted right intertrochanteric fracture, leading to her admission for surgical intervention.

Her past medical history was complex. She had undergone a hysterectomy for uterine fibroids ten years prior and a thyroidectomy for thyroid cancer 4 years ago, with current levothyroxine replacement (62.5 µg daily). She also had a history of leukopenia. Critically, she had experienced 2 recent episodes of spontaneous left pneumothorax, both managed with closed thoracostomy within the month preceding her admission, during which pulmonary nodules were documented. Furthermore, she had congenital spinal dysraphism (spina bifida) that was surgically repaired in childhood.

### 2.2. Physical examination

Upon admission, the patient was alert and oriented. Vital signs in the ward were stable, with a blood pressure of 138/68 mm Hg. Inspection of the back revealed a well-healed transverse surgical scar in the lumbosacral region. The spinous processes below the L3 level were palpably indistinct, although active lumbar spine range of motion was preserved. A significant deformity was noted in the left knee. Both ankles showed fixed fusion and dysplastic changes, consistent with her prior bilateral ankle arthrodesis. Neurological examination demonstrated symmetrically diminished sensation to light touch and pinprick below the knees bilaterally. Motor function in the lower extremities was limited due to the preexisting deformities and contractures, but she retained the ability to move her toes. The patient maintained full independence in activities of daily living, including self-care for urination and defecation.

### 2.3. Imaging findings

**Figure 1.**
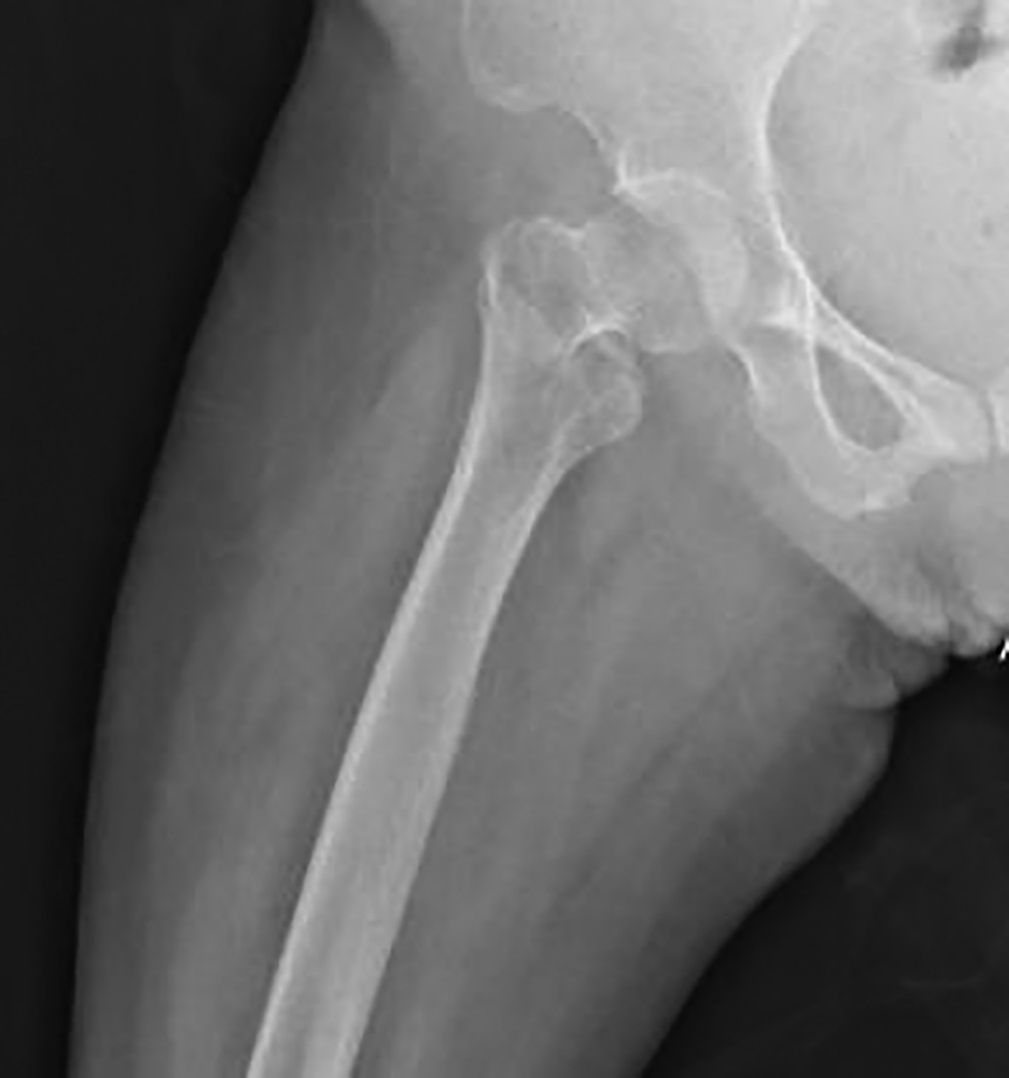
Coronal CT reconstruction of the right intertrochanteric fracture. CT = computed tomography.

Preoperative imaging from the current admission was pivotal for surgical and anesthetic planning. A non-contrast CT scan of the right hip confirmed a comminuted and displaced intertrochanteric fracture, as shown in Figure 1.

Given the patient’s complex medical background, previous imaging records were reviewed. Chest radiographs from her hospitalization one month prior documented a significant left-sided pneumothorax. A follow-up film obtained shortly before the current admission demonstrated successful resolution, showing no active pneumothorax at presentation.

**Figure 2.**
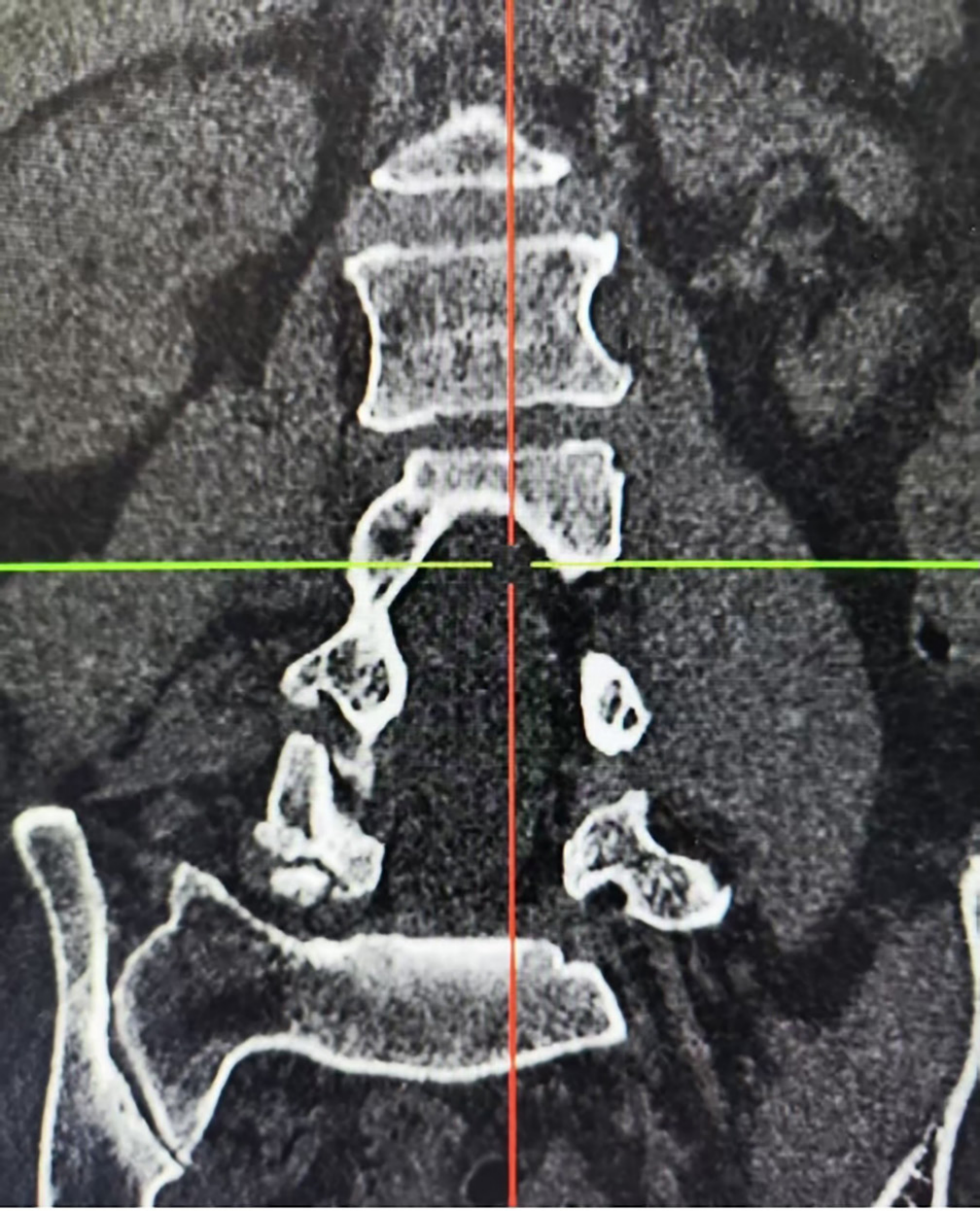
CT plain scan of the entire lumbar vertebrae. CT = computed tomography.

Comprehensive spinal imaging was performed during this admission to evaluate the anatomy for anesthetic planning. A lumbar spine CT scan revealed discontinuity of the spinous processes and laminae from L3 to S2, associated with a local meningocele, consistent with the known spinal dysraphism. The specific situation can be seen in Figure 2. A subsequent lumbar magnetic resonance imaging (MRI) provided further soft-tissue detail, confirming a Grade I spondylolisthesis of L5 and illustrating the postsurgical anatomical distortions. These findings unequivocally confirmed the high risk and technical infeasibility of neuraxial anesthesia.

### 2.4. Anesthetic management and intraoperative course

**Figure 3.**
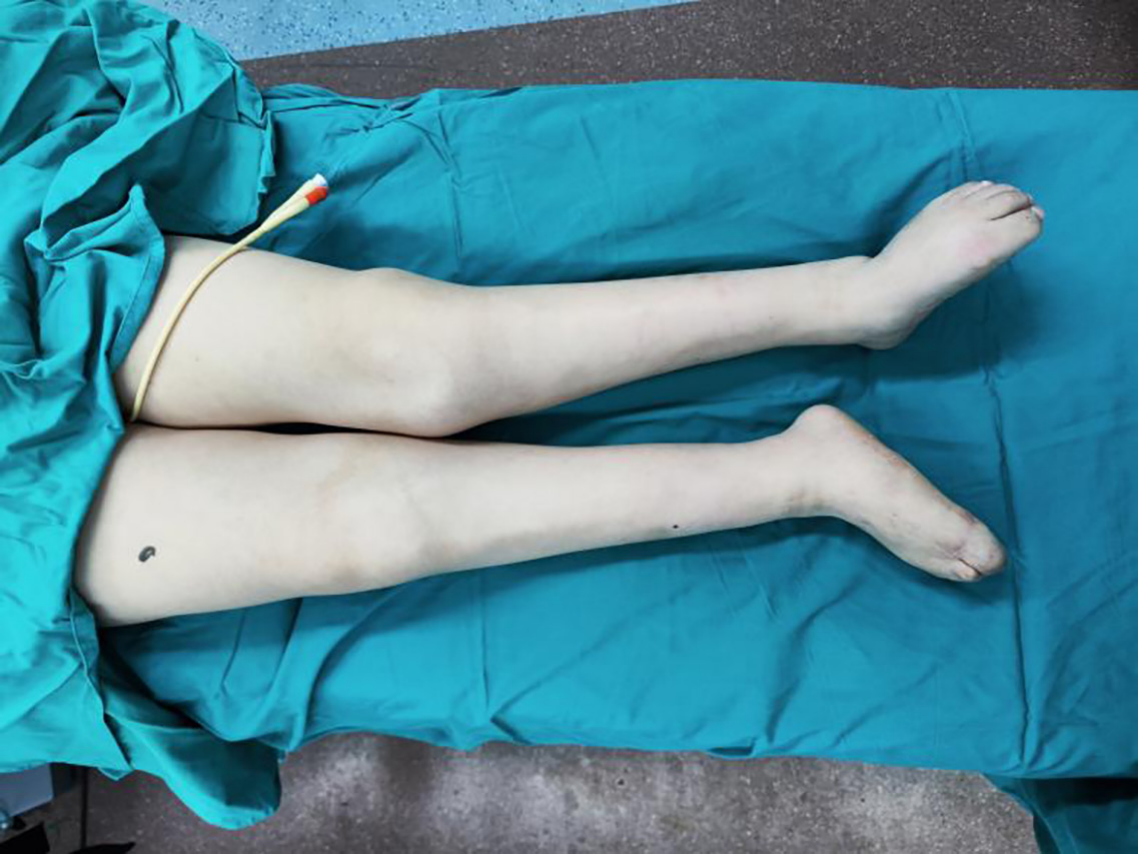
Appearance of the patient’s lower limbs before surgery. Transverse ultrasound image obtained with the probe placed parallel to the inguinal ligament. Labeled structures: external oblique muscle (EO), internal oblique muscle (IO), transversus abdominis muscle (TA), iliacus muscle (IM). ASIS = anterior superior iliac spine.

The patient was scheduled for closed reduction and internal fixation of the right intertrochanteric fracture on September 8. The preoperative lower limb condition of the patient is shown in Figure 3.

#### 2.4.1. Pre-anesthetic evaluation and decision-making

This study was approved by the Medical Research Ethics Committee of Shijiazhuang People’s Hospital (Approval No. 2025-177). A comprehensive anesthetic plan was formulated after considering the patient’s complex comorbidities. The congenital spinal dysraphism with distorted lumbar anatomy and preexisting neurological deficits represented a relative contraindication to neuraxial anesthesia. Furthermore, the 2 recent episodes of spontaneous pneumothorax created a significant concern regarding the risks of positive pressure ventilation during general anesthesia, a concern strongly echoed by the patient’s family. After multidisciplinary discussion and detailed patient consent, the decision was made to proceed with a primary ultrasound-guided nerve block technique, supplemented by intravenous analgesia and sedation, with a contingency plan to convert to general anesthesia with preserved spontaneous ventilation if necessary.

#### 2.4.2. Monitoring and anesthetic induction

Upon arrival in the operating room, standard monitoring was established, including noninvasive blood pressure, electrocardiography, and pulse oximetry. An invasive radial arterial line was placed for precise blood pressure monitoring, which initially read 203/88 mm Hg, attributed to procedure-related stress and anxiety (ward baseline: 138/68 mm Hg). After a period of reassurance and stabilization, the pressure decreased to 176/71 mm Hg. Given the patient’s recent history of recurrent spontaneous pneumothorax, a sedation strategy that preserves spontaneous ventilation was prioritized to avoid the risks associated with positive pressure ventilation. For respiratory monitoring, oxygen saturation was continuously monitored via pulse oximetry, and supplemental oxygen was administered at 3 L/min via nasal cannula. End-tidal carbon dioxide was monitored using a nasal sampling cannula. A bag-valve-mask device, anesthesia machine, and suction were prepared at the bedside for contingency, with the intention of avoiding positive pressure ventilation unless absolutely necessary.

#### 2.4.3. Nerve block technique

**Figure 4.**
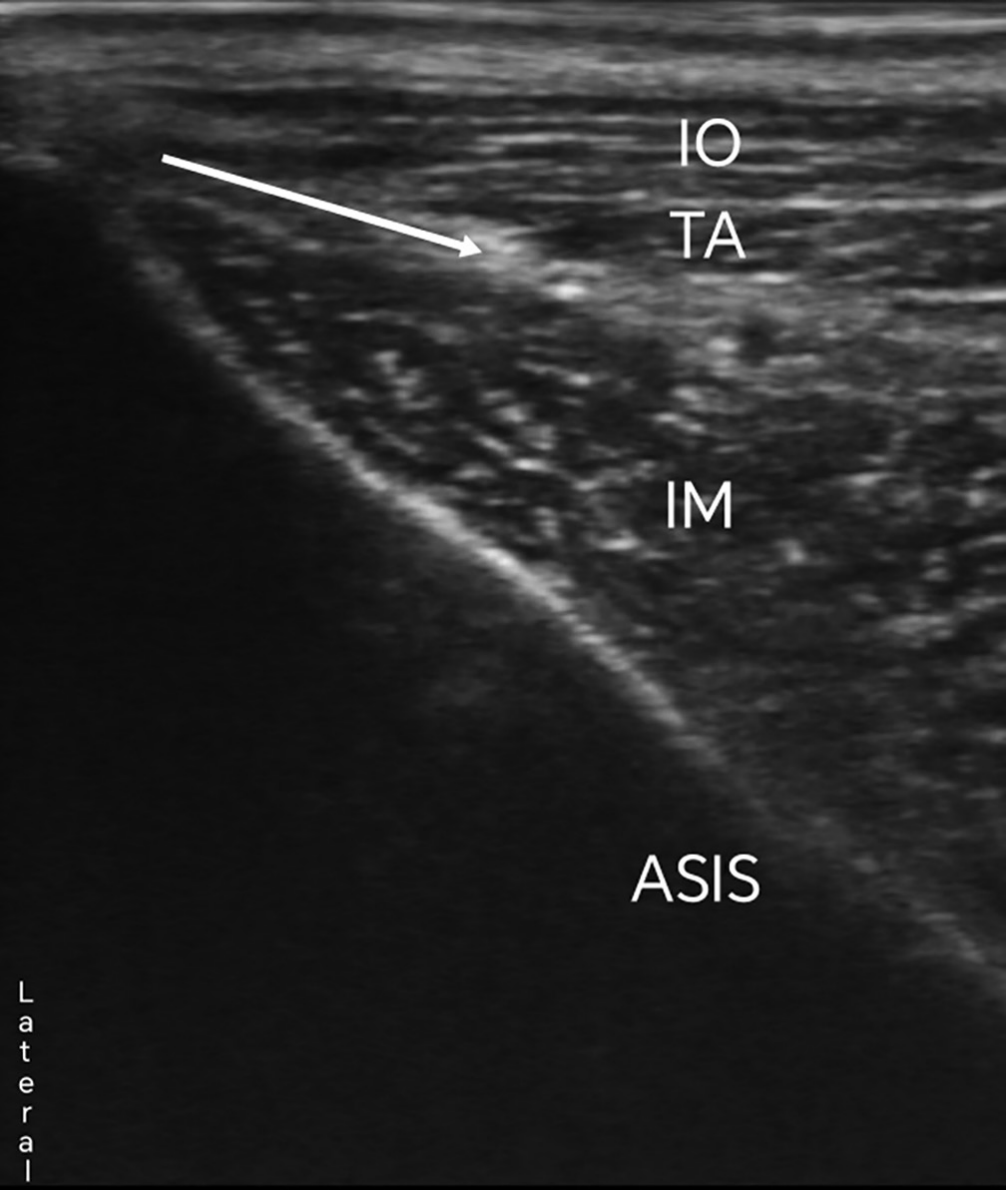
Ultrasound-guided supra-inguinal fascia iliaca compartment block. Transverse ultrasound image obtained with the probe placed at the level of the anterior superior iliac spine. Labeled structures: tensor fasciae latae muscle (TFLM), lateral femoral cutaneous nerve (LFCN), and sartorius muscle (SaM).

**Figure 5.**
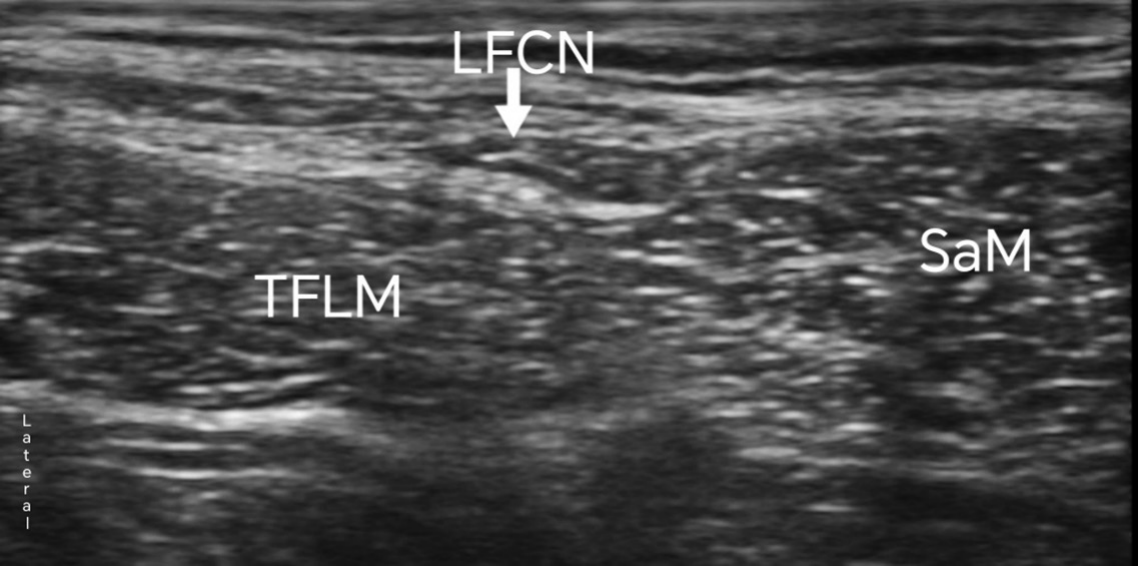
Ultrasound image of lateral femoral cutaneous nerve block.

Under full aseptic conditions and ultrasound guidance, a supra-inguinal FICB was performed, administering 40 mL of 0.4% ropivacaine (total dose 160 mg). The relatively large volume (40 mL) was used to increase the success rate of obturator nerve blockade. This dose remains within the safe upper limit for FICB (≤200 mg) as recommended by the Chinese Regional Anesthesia Guidelines and the American Society of Regional Anesthesia. The concentration of 0.4% was chosen to balance volume for adequate fascial plane spread and concentration for sufficient sensory blockade, given the patient’s low body weight (45 kg) and the need for reliable surgical anesthesia. No signs of local anesthetic systemic toxicity (LAST) were observed during the procedure. The specific situation is shown in Figure 4. To account for potential anatomical variation of the lateral femoral cutaneous nerve (LFCN), a separate targeted block of the LFCN was performed using 5 mL of 1% lidocaine. An additional 5 mL of 1% lidocaine was used for local infiltration along the planned surgical incision, given its proximal location over the right proximal femur. Thus, a total of 10 mL of lidocaine was administered for combined LFCN block and incision site infiltration. The specific situation is shown in Figure 5. The block was successful, resulting in evident sensory decline and numbness over the anterior and lateral aspects of the thigh.

#### 2.4.4. Intraoperative management

The patient tolerated positioning and limb traction without discomfort. The surgical procedure consisted of closed reduction and intramedullary nailing for the right intertrochanteric fracture. After positioning on a traction table under fluoroscopic guidance, traction was applied for approximately 15 minutes to achieve reduction. The procedure was performed through a longitudinal incision approximately 5 cm in length over the proximal right femur. Reaming was performed in a stepwise manner: proximal reaming was carried out to a canal diameter of approximately 2.5 cm, and distal reaming was performed with sequential increases in reamer size (ranging from 0.5 mm to 2.0 mm). However, upon initial surgical incision, the patient exhibited signs of discomfort (grimacing), reporting a verbal pain score (VAS) of 2/10. Surgical activity was paused immediately. A bolus of 11 mg esketamine was administered to provide immediate systemic analgesia, followed by a loading dose of remimazolam (0.1 mg/kg) administered slowly over one minute to achieve comfortable sedation while avoiding transition to general anesthesia. A continuous infusion of remimazolam at 0.25 mg·kg^-1^·h^-1^ was then initiated for maintenance. The patient fell asleep within 1 to 2 minutes, and the surgery proceeded uneventfully to completion, with a total duration of 70 minutes. No episodes of hypoxemia (peripheral oxygen saturation < 90%) or apnea occurred during the procedure, and spontaneous ventilation was maintained throughout.

Hemodynamics remained remarkably stable throughout: systolic blood pressure ranged from 128 to 151 mm Hg, and heart rate ranged from 61 to 78 bpm. The lowest blood pressure was observed after the onset of sleep but required no pharmacological intervention. The remimazolam infusion was discontinued at the end of surgery, and the patient awakened fully within 3 to 4 minutes, with blood pressure returning to pre-operative levels. She was transferred to the ward in a comfortable and stable condition.

#### 2.4.5. Postoperative outcome

**Figure 6.**
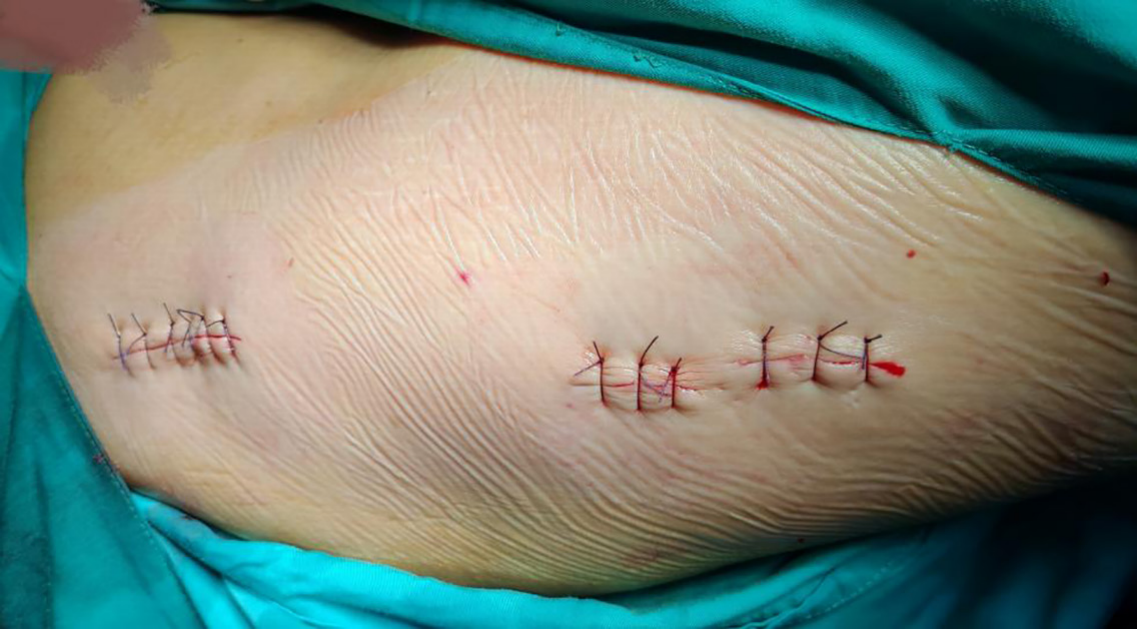
Post-surgical incision image.

Postoperative pain management consisted of intravenous ketorolac tromethamine (30 mg twice daily) for 3 days, during which the patient’s pain was well-controlled (VAS 1/10). The patient reported good sleep and mental status. She was discharged one week post-surgery without complications. A one-month telephone follow-up confirmed an unremarkable recovery. The incision situation after surgery is shown in Figure 6.

## 3. Discussion

This case report illustrates the successful implementation of a novel multimodal anesthetic technique, using a ultrasound-guided supra-inguinal FICB as the primary surgical anesthetic, supplemented with remimazolam-based sedation and esketamine analgesia, in a high-risk patient with relative contraindications to both neuraxial and general anesthesia. To our knowledge, this is one of the first reports detailing such a comprehensive “block-based” anesthetic strategy for intertrochanteric fracture surgery in a patient with this specific combination of comorbidities. Our experience demonstrates that this approach is not only feasible but also offers significant advantages in maintaining hemodynamic stability and facilitating rapid recovery.

The anesthetic management of intertrochanteric fractures in patients with significant comorbidities remains a subject of debate. While neuraxial anesthesia is often preferred for its potential benefits on mortality and thromboembolic events, its application is precluded in cases of congenital spinal abnormalities, previous spinal surgery, or coagulopathy.^[7]^ In our patient, the complex post-surgical anatomy and the presence of a meningocele, as confirmed by CT and MRI, rendered neuraxial techniques both technically challenging and potentially hazardous. Concurrently, the option of general anesthesia was deemed high-risk due to the patient’s recent recurrent spontaneous pneumothoraces. Positive pressure ventilation carries a non-negligible risk of pneumothorax recurrence or barotrauma in such individuals.^[8]^ The presented multimodal strategy effectively circumvented these dual risks.

The cornerstone of our technique was the comprehensive sensory blockade of the proximal femur. The supra-inguinal approach to the FICB has been shown to provide a more reliable spread of local anesthetic to the femoral, lateral femoral cutaneous, and obturator nerves compared to the classic infra-inguinal approach, making it superior for surgical anesthesia of the hip.^[9,10]^ Our proactive decision to perform a separate block of the LFCN underscores the importance of ensuring complete analgesia, given the known anatomical variability of this nerve. The initial success of the block was evidenced by the patient’s tolerance to positioning and traction. However, regional anesthesia for trochanteric fracture surgery ideally requires comprehensive coverage of the femoral, lateral femoral cutaneous, obturator, and sciatic nerves. Although the supra-inguinal FICB reliably anesthetizes the femoral and LFCNs, obturator nerve blockade is variable, and sciatic nerve blockade cannot be achieved via this compartment. Intraoperative fracture reduction and intramedullary instrumentation elicit profound nociceptive stimulation in structures innervated by these unblocked sciatic and obturator branches. Therefore, the breakthrough pain observed during surgical manipulation highlighted these predictable anatomical limitations. The supplemental administration of an 11-mg bolus of esketamine was crucial; it acted not merely as a sedative adjunct, but as a potent systemic analgesic that effectively countered the intense surgical stimuli, ensuring the successful completion of the surgery without conversion to general anesthesia.

While lumbar plexus block could theoretically achieve comparable surgical anesthesia with lower local anesthetic volume, it was not selected in this case due to specific safety considerations. The patient’s congenital spinal dysraphism and distorted lumbar anatomy (confirmed by CT and MRI) posed significant technical challenges and increased the risk of inadvertent neuraxial or epidural spread. Additionally, lumbar plexus block carries risks of retroperitoneal hematoma, nerve injury, and LAST. In the event of LAST, conversion to general anesthesia with positive pressure ventilation would be necessary, which was particularly undesirable given the patient’s recent history of spontaneous pneumothorax. The chosen strategy – supra-inguinal FICB combined with a separate LFCN block – provided effective surgical anesthesia while minimizing these specific risks.

The choice of sedative agents played a pivotal role in the success of this technique. Remimazolam, a newer ultra-short-acting benzodiazepine, offers unique advantages for procedural sedation, including rapid onset, context-sensitive half-life independent of infusion duration, and availability of a specific reversal agent (flumazenil).^[11]^ Its favorable hemodynamic profile was clearly demonstrated in our case, where blood pressure and heart rate remained remarkably stable throughout the procedure. The supplemental use of esketamine, an N-methyl-D-aspartate receptor antagonist, provided profound analgesic and sedative effects without respiratory depression.^[12]^ Its sympathomimetic properties may have further contributed to cardiovascular stability. This drug combination allowed for a state of comfortable sedation while preserving spontaneous respiration, which was critical for a patient with a recent pneumothorax history.

The combination of remimazolam and esketamine represents a rational synergistic approach for sedation in high-risk patients. Remimazolam, an ultra-short-acting benzodiazepine, provides rapid onset and recovery with reversal by flumazenil, while esketamine offers potent analgesia with preserved respiratory drive and fewer psychomimetic effects than racemic ketamine.^[13,14]^ This combination offers several advantages in the present case: preserved spontaneous ventilation avoids the risks of positive pressure ventilation, given the patient’s pneumothorax history^[8]^; hemodynamic stability is maintained through remimazolam’s minimal vasodilatory effects and esketamine’s sympathomimetic properties; and the availability of flumazenil provides an additional safety margin. Potential disadvantages include esketamine-associated psychomimetic effects (e.g., hallucinations, agitation), though these are dose-dependent and can be mitigated by benzodiazepines such as remimazolam.^[15]^ While this combination is not novel per se, its application as a rescue strategy in a patient with dual contraindications to conventional anesthesia – avoiding both neuraxial manipulation and positive pressure ventilation – represents the key clinical significance of this report.

The implications of this technique extend beyond merely overcoming anesthetic contraindications. For the growing population of frail elderly patients with hip fractures, anesthesia that minimizes physiological trespass is paramount. This block-centric approach attenuates the surgical stress response, provides superior postoperative analgesia, reduces opioid consumption and its associated side effects, and may facilitate earlier mobilization.^[16]^ All these factors are crucial elements of enhanced recovery after surgery protocols in orthopedic trauma.^[17]^

It should be emphasized that the anesthetic technique described in this report serves as a salvage strategy for high-risk patients rather than a routine substitute for standard neuraxial or general anesthesia. For patients without such complex contraindications, neuraxial or general anesthesia remains the standard of care supported by established clinical evidence. Without this clarification, readers might inadvertently conclude that the supra-inguinal FICB alone provides adequate surgical anesthesia for trochanteric fracture surgery. Because this regional technique carries an inherent risk of incomplete analgesia during deep tissue manipulation and fracture reduction, it demands meticulous patient selection, advanced block proficiency, and immediate readiness for airway intervention. In patients with congenital spinal dysraphism, neuraxial anesthesia carries risks of technical failure, neurological injury, or exacerbation of preexisting deficits. Similarly, general anesthesia with positive pressure ventilation poses a non-negligible risk of pneumothorax recurrence in patients with recent spontaneous pneumothorax. When both standard options are relatively contraindicated, a block-based strategy supplemented by sedation with agents that preserve spontaneous ventilation and hemodynamic stability becomes a rational alternative. However, this approach demands rigorous patient selection, meticulous block execution, and continuous monitoring with contingency plans for airway management. In patients without such contraindications, neuraxial or general anesthesia remains the standard of care based on established evidence.

## 4. Conclusions

In conclusion, for patients with intertrochanteric fractures and relative contraindications to conventional anesthetic techniques, a primary multimodal approach centered on a comprehensive ultrasound-guided nerve block, supplemented by light sedation with remimazolam and esketamine, represents a viable, safe, and effective salvage strategy. This approach should be reserved for carefully selected high-risk patients in whom standard neuraxial or general anesthesia is deemed relatively contraindicated. When no such contraindications exist, conventional anesthetic techniques remain the standard of care based on their established safety and efficacy profiles.

## 5. Patient perspective

The patient was consulted preoperatively and reported significant anxiety, primarily concerning the risks associated with anesthesia, given her complex medical history. Following a detailed explanation of the proposed nerve block-based technique, she provided informed consent. Postoperatively, the patient reported a positive recovery experience. She recalled the procedure as being comfortable and expressed satisfaction with her rapid awakening and clear-headedness after surgery. At the one-month follow-up, she confirmed a smooth convalescence without complications.

## Author contributions

**Conceptualization:** Xiaolin Wang, Yanchao Yang.

**Data curation:** Xiaolin Wang, Jingjing Zhang, Jing Ren, Ya Liu, Yanchao Yang.

**Formal analysis:** Jingjing Zhang, Jing Ren, Ya Liu.

**Investigation:** Xiaolin Wang, Jingjing Zhang, Yanchao Yang.

**Methodology:** Xiaolin Wang, Jingjing Zhang, Jing Ren, Ya Liu, Yanchao Yang.

**Writing – original draft:** Xiaolin Wang, Yanchao Yang.

**Writing – review & editing:** Xiaolin Wang, Jingjing Zhang, Jing Ren, Ya Liu, Yanchao Yang.
